# Oxidation of Drugs during Drug Product Development: Problems and Solutions

**DOI:** 10.3390/pharmaceutics14020325

**Published:** 2022-01-29

**Authors:** Alen Gabrič, Žiga Hodnik, Stane Pajk

**Affiliations:** 1 Krka d.d., R&D, Šmarješka Cesta 6, 8001 Novo Mesto, Slovenia; alen.gabric@krka.biz; 2 Faculty of Pharmacy, University of Ljubljana, Aškerčeva Cesta 7, 1000 Ljubljana, Slovenia

**Keywords:** oxidation, peroxides, impurities, new oxidative stressors, oxidative stress testing

## Abstract

Oxidation is the second most common degradation pathway for pharmaceuticals, after hydrolysis. However, in contrast to hydrolysis, oxidation is mechanistically more complex and produces a wider range of degradation products; oxidation is thus harder to control. The propensity of a drug towards oxidation is established during forced degradation studies. However, a more realistic insight into degradation in the solid state can be achieved with accelerated studies of mixtures of drugs and excipients, as the excipients are the most common sources of impurities that have the potential to initiate oxidation of a solid drug product. Based on the results of these studies, critical parameters can be identified and appropriate measures can be taken to avoid the problems that oxidation poses to the quality of a drug product. This article reviews the most common types of oxidation mechanisms, possible sources of reactive oxygen species, and how to minimize the oxidation of a solid drug product based on a well-planned accelerated study.

## 1. Introduction

Safety and efficacy are key aspects of drug research and development, therefore, formulations must be designed in a way that ensures appropriate bioavailability of a drug and its physico-chemical stability over the determined shelf-life [1]. The chemical stability of a drug is an intrinsic property that is determined by its chemical structure. The dosage form can lead to drug instability because of the presence of other compounds (e.g., excipients). Also, the drug manufacturing process, packaging, and storage must be monitored for drug stability. In this context, the problem of drug product stability is an very important area in drug research and development, not only for new drugs, but also for generic drugs [2,3].

A drug product can undergo physical and chemical changes. The first affects the form of the chemical substance but not its chemical composition, which means that no chemical bonds are broken or formed [4]. The physical instability of a drug manifests as changes in its appearance, the release of the drug, polymorphic changes, adsorption, and many others [5]. On the other hand, chemical changes refer to changes in the chemical structure that arise from degradation of the drug substance and the reactions between the drug and the excipients in the formulation. These changes can reduce the drug potency, which raises efficacy concerns, and, at the same time, they can pose a safety risk, as the degradation products might be toxic.

One of the first steps in the development of any new drug is a forced degradation study, where the first insights into its chemical stability are obtained. Before the start of the forced degradation study, the predominant degradation products can be predicted by in silico tools such as Zeneth [6]. Zeneth operates on the basis of rules of chemical transformations that are written down with Markush structures. When the program recognizes structural patterns in the molecule it applies transformation rules and converts the molecule into its corresponding products [7]. The structures of the degradation products are established primarily using liquid chromatography–high resolution mass spectrometry (LC-HRMS), when this is not possible (e.g., ionization problems, volatility) other techniques are used, such as gas chromatography–mass spectrometry (GC-MS), evaporative light scattering detector (ELSD), and charged aerosol detector (CAD); however, if necessary, other methods can be used to further confirm the structures (e.g., nuclear magnetic resonance) [8].

One of the most important steps after identifying new degradation products is the risk assessment for potentially mutagenic compounds. The ICH M7 (assessment and control of DNA reactive (mutagenic) impurities in pharmaceuticals to limit potential carcinogenic risk) guideline is written on this topic [9], which describes, in detail, the process of toxicity assessment (the use of two complementary in silico systems, for e.g., DEREK and Leadscope) [10,11], setting limits (threshold of toxicological concern (TTC) or median toxic dose (TD_50_) [12,13]), and analytical control (developing analytical methods).

**Figure 1:**
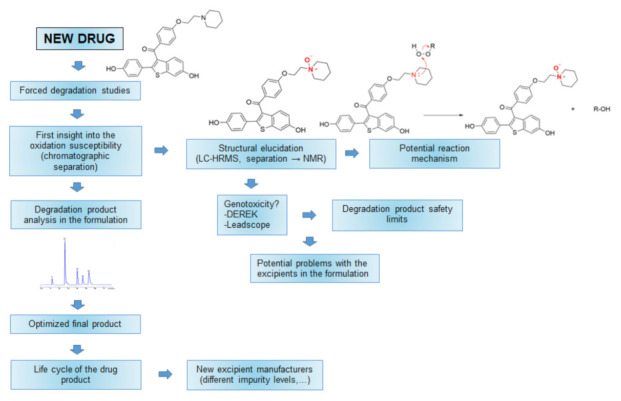
Schematic representation of oxidation reactions and their role through drug development. Of note, in this review the word “drug” corresponds to “active pharmaceutical ingredient” and not to “final drug product”.

With the information from the forced degradation study, researchers can evaluate the stability of a drug under different conditions according to the amounts of degradation impurities that are formed. After the structures of the degradation products have been determined, there is the need to determine what types of chemical reactions are involved in the degradation of the drug (Figure 1). This information helps to predict the reactions that can occur with excipients and their contaminants [14].

For drugs that are susceptible to oxidation, compatibility with some excipients might become a problem. With the knowledge of the degradation products, a stability-indicating method can be developed, which is the starting point for the development of the final methods for the analysis of a formulation [15]. Drug formulations are continuously analyzed during their development, with a specific focus on the stability of the drug. This information, in turn, influences the selection of suitable excipients until adequate stability of the formulation is obtained, e.g., the addition of antioxidants for oxidation-susceptible compounds. Even after the approval of a drug product, the suppliers of the excipients might change during its life cycle or different qualities of excipients might be used [16]. All of these changes should be closely monitored from the analytical point of view, as certain degradation impurities might increase.

**Figure 2:**
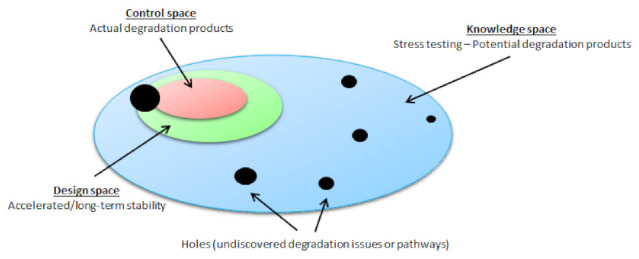
Conceptual diagram of quality by design, showing the relationships between the knowledge space, design space, and control space.

All of the knowledge that we gain during stress testing can be presented as the ‘knowledge space’, which is a concept that comes from quality by design (QbD). The integrity of the knowledge space depends on the scientific validity and quality of the research. In summary, stress testing should identify all reasonably possible degradation products that can occur under real-world conditions. All of these products are treated as potential degradation products. The actual degradation products that are formed from a certain drug, however, will depend on the physical conditions, dosage forms, excipients, and packaging, and should be a subset of the potential degradation products. According to QbD terminology, the ‘design space’ lies within the knowledge space, as a combination of the previously mentioned variables, which, in this case, represent the degradation products that are formed during accelerated or long-term stability studies. Then, within the design space, there is the ‘control space’ which represents the optimum of all of the variables for optimal stability. Incomplete, poorly designed, or poorly performed stress tests can result in a lack of detection of significant degradation pathways. The result of this is an incomplete understanding of the degradation chemistry of the drug. These missing pieces of information are represented as holes in the knowledge space (Figure 2). Therefore, it is important to understand what some of these holes are so that they can be avoided [17,18,19].

There are numerous types of chemical reactions that can affect a drug; however, in this review, we focus on oxidation during drug product development. Oxidation is the second most frequent degradation pathway for pharmaceuticals after hydrolysis, and it is, therefore, important to understand the underlying mechanisms to be able to formulate effective prevention strategies [20,21].

## 2. Oxidation Reactions

The following primary oxidative degradation mechanisms are known:

- Autoxidation (radical mediated);
- Nucleophilic/electrophilic (peroxide mediated);
- Oxidation that is mediated by single electron to dioxygen.

### 2.1. Autoxidation

The term autoxidation classifies the oxidation of a substrate by molecular oxygen, ^3^O_2_. Autoxidation can start a chain process when the oxidized substrate generates a reactive species that subsequently attacks additional substrate molecules. This mechanism is also known as a radical chain reaction, where the addition of oxygen gives rise to hydroperoxides and their associated peroxy radicals (ROO^•^) [22,23]. The radical chain reaction is referred to as the Bolland–Gee mechanism [24].

At this point, we note that according to the recommendations in this review, we do not use the term free radical. In the context of physical organic chemistry, it appears desirable to cease using the adjective ‘free’ in the general name of this type of chemical species and molecular entities, so that the term ‘free radical’ might in the future be restricted to those radicals that do not form parts of radical pairs [25].

**Figure 3:**
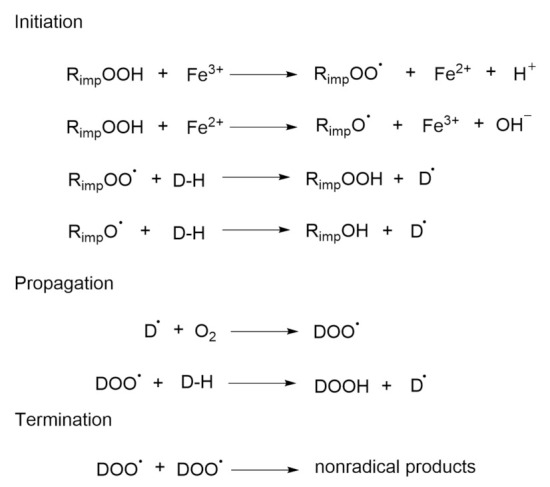
Autoxidation that is mediated by a drug-based peroxy radical (DOO^•^).

Chain processes consist of three concurrent reactions: initiation, propagation, and termination [26] (Figure 3). Depending on the radical concentrations and the specific rate constants, each of these reactions will dominate within a particular time domain, which leads to the three distinct phases. The initiation step requires a radical initiator that generates radicals [27]. The initiation reaction can be triggered by hydroperoxides (ROOH), which are common excipient impurities [28].

The initiation of oxidation reactions can involve the abstraction of H-atoms from various moieties of the drug substance by the impurity-derived radicals. These can result from the reaction between hydroperoxides and trace amounts of iron or copper ions, whereby the result of both of these reactions is the same: the drug radical (D^•^), here, copper, is about 50 times more reactive than iron. This is followed by the reaction between oxygen from the air and D^•^, which produces drug-derived peroxy radicals (DOO^•^), which can abstract hydrogen from another drug molecule. This is the key reaction in autoxidation, and once it takes place, the chain reaction is set. The chain can be relatively long, where only one initial radical is needed to produce hundreds of hydroperoxide molecules. Reactions that are involving peroxy radicals are relatively slow, so this is the limiting step in the chain, and peroxy radicals might start to accumulate. The hydroperoxide (DOOH) that is formed is the first stable oxidation product that can be identified [29].

**Figure 4:**
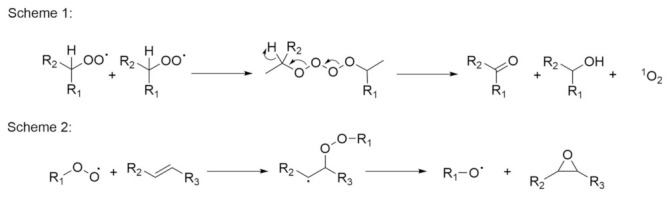
The termination reaction (Scheme 1) and epoxide formation (Scheme 2).

The last step in the chain reaction (i.e., termination) occurs when the amount of the drug is reduced to such an extent that the peroxy radicals begin to react with each other. The reaction product of these two radicals is a nonradical. If, in this last reaction, two molecules of peroxy radicals react with each other, a tetroxide is formed, which decomposes into an alcoholic moiety and a carbonyl, as in Scheme 1 (Figure 4) [30]. Peroxy radicals can also react with double bonds (carbon-carbon) during the propagation phase, to form epoxides, as in Scheme 2 (Figure 4) [31].

In the 1990s, Boccardi claimed that, through testing, he had found that most oxidation in pharmaceuticals are the result of autoxidation [32,33]. This finding was not surprising, because, as we will see below, organic hydroperoxides are present in many commonly used excipients. Likewise, metals, such as iron and copper, are ubiquitous as they cannot be completely removed during or after drug and excipient synthesis. Thus, all of the ingredients to start autoxidation are present in most formulations. The main reason why autoxidation is not more problematic is due to the low sensitivity of some drugs towards oxidation, and, even if oxidation does occur, the safety limits for degradation products are, in some cases, set relatively high, on a parts per million (ppm) scale.

### 2.2. Nucleophilic/Electrophilic (Peroxide Mediated)

Peroxide-mediated reactions are the second most common oxidation mechanism after autoxidation, where a drug reacts with hydrogen peroxide. Peroxides are present in commonly used excipients, and, because of this, these reactions occur in most formulations with drug substances that are susceptible to oxidation. These reactions are slow and occur under long-term storage. Hydrogen peroxide reacts with secondary and tertiary amines, thioethers, and olefins. Examples of each reaction with hydrogen peroxide are given below [34,35,36].

As indicated, hydrogen peroxide can react with both secondary and tertiary amines, but the reaction is more favorable with tertiary amines. The reaction is also pH-dependent as it is slower in the protonated state. In solids, the same concept applies where drugs in the form of salts can disproportionate to the more oxidation-prone non-ionized form [37]. Disproportionation in solids most often occurs during processing, e.g., in wet granulation, drying, tableting, or during storage, because of the excipients in the formulation [38]. Excipients affect disproportionation in two ways; firstly, by creating a pH microenvironment and secondly, with their buffering capacity. When disproportionation happens, the drug remains in a non-ionized form which means it becomes susceptible to oxidation [38,39,40].

**Figure 5:**
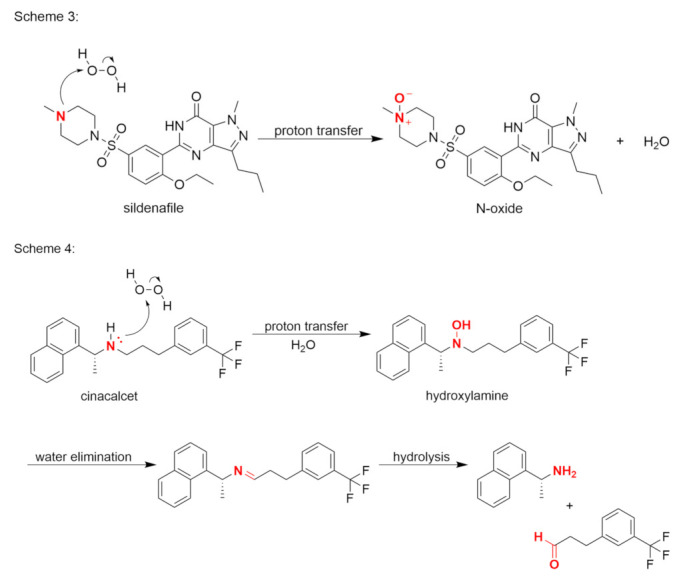
Formation of an N-oxide (Scheme 3) and a hydroxylamine, with hydrolysis (Scheme 4).

A good example is the reaction between tertiary amine and hydrogen peroxide in sildenafil. The methylpiperazine moiety reacts with hydrogen peroxide to form the N-oxide sildenafil impurity, as shown in Scheme 3 (Figure 5) [41]. Scheme 4 shows an example of cinacalcet, a secondary amine, where a hydroxylamine is formed in the first step (Figure 5). After the elimination of water, the enamine is hydrolyzed to form a primary amine and an aldehyde [42].

**Figure 6:**
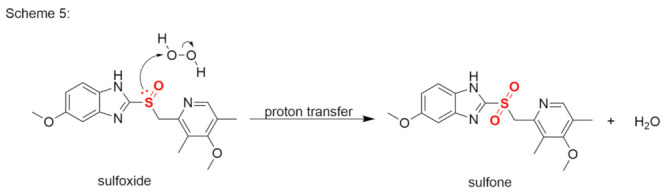
Reaction between a sulfoxide and hydrogen peroxide (Scheme 5).

The reaction between a thioether and hydrogen peroxide produces a sulfoxide in the first step, and a sulfone in the second. The sulfone-production reaction is shown in Scheme 5 (Figure 6), using omeprazole as an example [43].

### 2.3. Oxidation Mediated by Single Electron Transfer to Dioxygen

**Figure 7:**
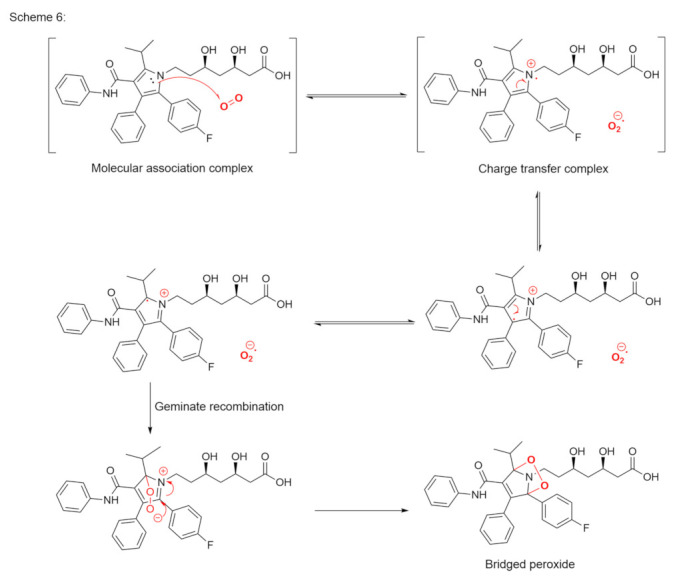
Proposed reaction mechanism for atorvastatin with the formation of a bridged peroxide.

While the vast majority of oxidation reactions can be described according to the two oxidation mechanisms above, certain compounds can undergo single electron transfer to dioxygen. Compounds that are susceptible to these reactions must contain a weakly acidic hydrogen atom, which, in a basic environment, is cleaved to form a carbanion [44]. The carbanion undergoes single electron transfer to oxygen, which forms a carbon radical and a superoxide radical. The final product of this reaction is the formation of a hydroperoxide anion. The reaction of carbanions with molecular oxygen has been studied in the context of base-catalyzed oxidation and has been extensively reviewed. Weakly acidic C-H bonds, such as aliphatic nitro compounds [45], triphenylmethane [46], ketones and esters [47], aryl propenes [48], and nitriles, can yield carbanions upon treatment with a base, where the carbanions that are formed can then rapidly react with dissolved oxygen. Scheme 6 shows a likely reaction mechanism for atorvastatin with the formation of a metastable bridged peroxide product (Figure 7) [49].

## 3. Drug Interactions with Excipient Impurities

In most cases, drugs are stable in their pure form, with instability arising due to their mixing with excipients. These excipients can contain trace level impurities which can then react with the drug. The most common impurities in excipients are peroxides; however, from the point of view of oxidation, metals are also important, even when they are present in trace amounts. In this section we will look at the most common impurities that can be found in excipients.

### 3.1. Peroxides

Peroxides can, in general, be either organoperoxides (ROOR′) or hydroperoxides (ROOH) [50]. All peroxides contain a very weak O–O bond, that can easily split and form hydroxyl (^•^OH) and alkoxy (RO^•^) radicals. Other reactive oxygen species, for example superoxide anion (O_2_^− •^), hydrogen peroxide (H_2_O_2_), and hydroperoxides can form from peroxides and radicals. As mentioned before, the stability of a drug product usually depends on what pharmaceutical excipients are being used, as they contain impurities, for example hydroperoxides and organoperoxides are the common peroxide impurities and can cause oxidative degradation of a drug [51].

Hydroperoxides are common trace level impurities in excipients such as polyethylene glycol (PEG), povidone, hydroxypropyl cellulose, and polysorbate [51]. All of these are polymeric excipients, where peroxides are used to initiate the polymerization reaction, which leaves trace levels of peroxides as a by-product [52]. It is then difficult to completely eliminate these from the final product. Recently, it has been suggested that the introduction of peroxides can occur after synthesis during the drying process, especially with the high temperatures that are used in these processes [53].

There are several techniques in the literature for quantifying trace levels of peroxides (e.g., hydrogen peroxide, organic peroxides) in the atmosphere and in biological samples, which include UV/fluorescence detection [54] and electrode-based sensors [55]. The reported UV detection methods typically require sample derivatization or manipulation steps that produce a chromophore. However, there are only a few studies that have described quantification of trace levels of hydrogen peroxide in pharmaceutical excipients (i.e., ppm). HPLC with diode-array detection is a more indirect method of determination which is based on oxidation of triphenylphosphine into triphenylphosphineoxide [56,57]. The ferrous oxidation–xylenol orange method with catalase is another indirect method which uses UV detection [51,58,59]. This method can be used to determine the amount of total peroxides, the amount of hydrogen peroxide, and the amount of organic peroxides. Huang et al. measured hydrogen peroxide in excipients using a direct method that combined liquid chromatography with amperometric electrochemical detection [60]. Hongfei et al. upgraded this method by converting it to coulometric electrochemical detection, while also modifying the chromatography to provide better separation between hydrogen peroxide and the excipients [61].

Wasylaschuk et al. analyzed 10 common pharmaceutical excipients from different lots to determine the hydroperoxide levels. Their reported values of the significant levels of hydroperoxides that were determined in common excipients provide a good insight into the range of the hydroperoxide contents that were found: polyvinylpyrrolidone (PVP): mean 250 ppm, highest 380 ppm, lowest 120 ppm; PEG 400: mean 75 ppm, highest 115 ppm, lowest 35 ppm; polysorbate 80: mean 50 ppm, highest 160 ppm, lowest 6 ppm; and hydroxypropyl cellulose: mean 10 ppm, highest 30 ppm, lowest 2 ppm. Other excipients contained <1 ppm hydroperoxides, which was also their limit of quantification. They also reported that the hydroxypropyl cellulose certificate does not contain hydroperoxide testing data. However, if the peroxide contents were given, these were indicated as lower than those that were determined here. Therefore, they also suggested that end users should actively monitor excipients using a single assay methodology [51]. Both hydroperoxides and hydrogen peroxide contribute to the determination of total peroxides in excipients; however, both do not necessarily oxidize the drug at the same rate, and, therefore, the total peroxides will also not give the whole picture.

At this point it should also be noted that even when oxidation of a drug is not a problem, the hydroperoxide levels in excipients can lead to other formulation challenges. Different studies have shown that hydroperoxide levels and the ‘oxidizability’ of excipients can lead to significant changes in pH, appearance, and viscosity [62,63,64].

### 3.2. Metals

**Figure 8:**
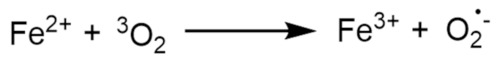
Proposed mechanism for metal ion reaction with triplet oxygen to form a superoxide.

Metals are ubiquitous in pharmaceutical excipients, and, at trace levels, they can catalyze the oxidation of pharmaceuticals. The reaction of molecular oxygen with most organic molecules is thermodynamically favored. However, the reaction of triplet state molecular oxygen (i.e., the ground state) and singlet molecules is spin-forbidden. Trace metal impurities can react with triplet oxygen to reduce a molecule to a more kinetically favored oxidizing agent, such as a superoxide (Figure 8) [65,66,67].

**Figure 9:**
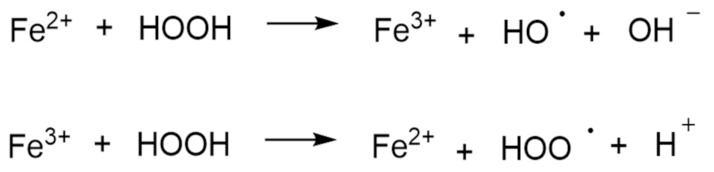
The Fenton reaction.

Another common mechanism, as mentioned above for autoxidation, involves Fenton-like reactions, where the oxidized or reduced form of a catalytic transition metal, such as Fe(III) or Fe(II), reacts with hydrogen peroxide to produce several more reactive species. Hydrogen peroxide can be reduced to a hydroxyl radical and hydroxide, or oxidized to a peroxy radical and a proton, as shown in Figure 9 [68,69].

For safety reasons, the metal content is highly controlled in drugs and excipients with low limits that are set by the regulatory guidelines [70]. Therefore, there are not many reported cases in the literature where the degradation of the drug is caused by a metal impurity.

## 4. Prevention

### 4.1. Peroxide Control Strategies

As mentioned earlier, the concentration of peroxides in excipients can vary from lot to lot and from manufacturer to manufacturer. The change in peroxide concentration during storage of excipients and drug products can lead to unpredictability of the drug product stability. Therefore, reducing the initial peroxide concentrations in excipients and maintaining low peroxide concentrations in the formulation can help to improve the stability of oxidation-labile drugs in their dosage forms [71]. Approaches to reduce peroxides in excipients include the use of enzymes and metals [72], chemical modification of cross-linkers [53], supercritical fluid extraction [73], and vacuum drying [74]. However, the peroxide levels in excipients often increases during storage, as seen for povidone, PEG, and others [75].

### 4.2. Storage Conditions

Narang et al. studied the influence of storage conditions on peroxide concentrations in povidone. Their data indicated that low humidity and high temperatures can lead to increased peroxide concentrations [71]. As expected, the mass of povidone increased at high humidity and decreased at low humidity, except at a relative humidity of 32%; this indicates that povidone should be stored under this condition [76]. However, the additional mass due to moisture absorption does not explain the change in peroxide concentration. The decrease in peroxide concentration at high humidity can be explained by water–peroxide exchange, quenching of the singlet oxygen trapped in povidone, and/or peroxide decomposition [77]. As mentioned, water can act as a quencher of the reactive singlet oxygen, thus minimizing oxidative radical reactions and the generation of peroxides. However, this process is slow and occurs on a very limited scale [78]. The uptake of water in povidone can increase the mobility of the molecules [79] which can increase the reactivity of the impurities in the povidone, to lead to peroxides consumption. The content of total peroxides is divided into hydrogen peroxide and hydroperoxides (ROOH). However, in the above example, the amount of hydrogen peroxide was negligible. Therefore, we can conclude that the peroxide content is related only to the hydroperoxide groups on the polymer itself. Although the exact reason for the reduction in peroxide concentration at high humidity remains unknown, studies have indicated that increased accessibility of ROOH might be the overriding mechanism for this reduction in the peroxide content in povidone [71]. Another reason why low peroxide content at high humidity was surprising was because the excipients are usually stored under dry conditions to ensure the physical stability of hygroscopic excipients, such as povidone. In addition, storage at very low humidity is often considered desirable for drug products to minimize hydrolytic and other drug degradation reactions that are attributable to direct reactivity with water. It is also worth noting that the process of peroxide formation was slowed down at 32% relative humidity, and hence this represents the recommended humidity for the storage of povidone.

### 4.3. Antioxidants

One of the approaches that is used in the drug product formulation is the use of antioxidants. Antioxidants are compounds that can prevent the oxidation of other molecules. Depending on their antioxidative mechanism of action [80], they can be:

(a) Initiation inhibitors. Antioxidants can prevent radical chain reaction as they react with initiators of radical chain reactions. The most common representative of this group is ethylenediaminetetraacetic acid (EDTA), which can also acts as a heavy-metal chelating agent [81]. It is also necessary to pay attention to the type of metal that is complexed with EDTA. If iron ions are present, the addition of EDTA produces an iron–EDTA complex that, counterintuitively, accelerates the formation of hydroxyl radicals. On the other hand, an iron complex with diethylenetriaminepentaacetic acid does not accelerate the Fenton reaction. However, all of the studies that are described here were performed in aqueous solutions, so without additional testing it is difficult to assume the same applies to solid dosage forms [82].
(b) Terminators of radicals. Some antioxidants, such as butylhydroxyanisole (BHA) and butylhydroxytoluene (BHT), can react with radicals, to thus inhibit the propagation phase of the radical chain reaction [83,84].

**Figure 10:**
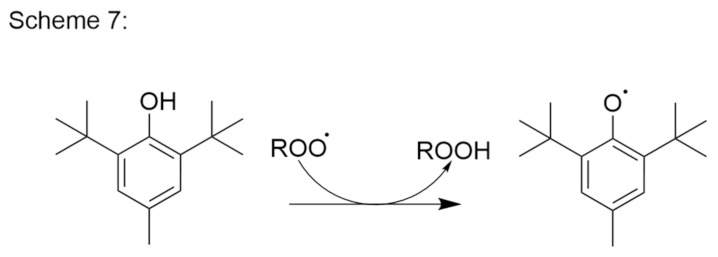
Butylhydroxytoluene reaction with the peroxy radical and stabilization of the radical electron.

In nature there is an abundance of phenolic compounds with the tendency to be relatively stable radicals after one-electron oxidation. With the addition of bulky alkyl groups to the aromatic ring, such as t-butyl in the case of BHA and BHT (Scheme 7, Figure 10), the stability of the radical electron increases. Such antioxidants tend to form relatively inert radicals, to thus terminate the chain reaction [83]. To prevent oxidative degradation of lovastatin in an aqueous solution, α-tocopherol is more effective than BHA, which, in turn, is more effective than propyl gallate [85].

**Figure 11:**
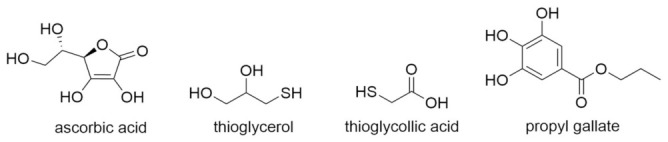
Reducing agents.

(c) Antioxidants as reducing agents. Antioxidants can act as reducing agents by being selectively oxidized, to thus protect the substrate by competitive reactivity. Ascorbic acid, thiols (e.g., thioglycerol, thioglycollic acid), and polyphenols (e.g., propyl gallate) can act as such reducing agents (Figure 11).

Narang et al. reported that the effectiveness of antioxidants for the prevention of oxidation is related to their hydrophilicity [86]. Studies have been performed both in aqueous solution and in the dry, and at different humidities. Of all of the antioxidants that were tested, BHT was the least effective. At first, this appears unexpected because BHT is the most widely used antioxidant in drug formulations. On the other hand, this is consistent with other literature sources [87]. For example, Smirnova et al. showed that under certain conditions, BHT can react with oxygen in an aqueous medium to form a reactive superoxide radical anion [88]. BHT was the only insoluble antioxidant among all of those that were tested, and insoluble antioxidants tend to be predominantly suspended as fine particles, which, in turn, reduces their reactivity with peroxides on the polymer excipient. Greater efficiency was also shown for ascorbic acid and propyl gallate than BHA and BHT, where these last two have just one hydroxyl group and higher lipophilicity [89].

Another approach that can be implemented if susceptibility to oxidation is found is to conduct a stability study. This is done with excipients that contain different concentrations of peroxides, or by spiking one lot of an excipient with different amounts of peroxide. For example, Hartauer et al. spiked raloxifene hydrochloride tablets with different quantities of hydrogen peroxide that were equivalent to 200 ppm to 800 ppm on top of that which was already present in povidone and crospovidone. With this methodology, a safe limit can be determined, below which negligible oxidation occurs [90,91].

### 4.4. Metal Control Strategies

Metal chelators are often used to scavenge reactive transition metals from formulations (see section on initiation inhibitors); however, hydrogen peroxide can often react with the chelator–metal complex to also generate reactive species [65]. The safe limit of peroxides can be set by carrying out an accelerated stability study where an excipient is spiked with metal ions [92]. In practice, metals are not that problematic, as they get removed with various extractions during the workup after the synthesis. Therefore, metals usually do not cause problems in the final pharmaceutical forms. At the same time, as it is stated, the addition of chelators may produce chelator-metal complexes that catalyze the decomposition of peroxides to reactive species, thus worsening the problem.

### 4.5. Packaging

For autoxidation instability processes where atmospheric oxygen is the source of the oxidant, the packaging can represent an option for the stabilization of the drug. However, the problem with packaging is that this must take into account both the oxygen that is available in the headspace and the oxygen that permeates through the container walls and caps. For most plastic containers, the rate of oxygen permeation can be significant. To prevent oxygen permeation into the container, the use of foil–foil blisters is necessary. Packaging must be carried out in an inert atmosphere (i.e., under nitrogen or argon), although this process can be cumbersome for solid dosage forms [93]. Reducing the headspace oxygen concentration and lowering the oxygen permeation does improve the stability, although significant protective benefits are seen only at very low oxygen concentrations. Packaging under internal conditions is, in most cases, the last option where problems with autoxidation cannot be solved [94,95].

## 5. Oxidative Susceptibility Testing

A forced degradation study defines the degradation of a drug under conditions that are more severe than accelerated conditions and includes oxidation stability testing. Forced degradation studies can provide the basic knowledge space of the chemical behavior of a drug, which helps in the development of its formulation and packaging [96]. Despite the importance for further development, the regulatory guidelines are very general and do not provide any strict protocols. The International Council for Harmonisation guidelines suggest that stress testing should be designed to identify likely degradation products, which helps to determine the intrinsic stability of a molecule and to predict its degradation pathways [3]. With the help of the degradation products, the indicated stability method can be validated, which will then be used in the development of the formulation [97]. Nevertheless, over the years, many trends have emerged on how to conduct degradation studies by proposing strategies for conducting studies with a focus on degradation mechanisms.

At the beginning of the research on chemical stability of a new molecule, the identification and characterization of its degradation products can also provide alerts for potential genotoxicity of certain degradation products and can influence how much effort is put into the control of such impurities and their acceptable limits. All of this information provides a better understanding of a drug, which, in turn, allows for more efficient development of its formulation [98,99].

The goal of susceptibility testing is to accelerate the oxidative processes that would otherwise occur over a long period of time. In this review, we systematically examine examples and methods of susceptibility testing in the same order as described in the section on oxidation reactions. These studies are designed to mimic normal long-term oxidation processes, and five goals can be identified for such testing [100]:

- Prediction of whether or not a drug is sensitive to oxidation;
- Determination which specific oxidative degradation mechanism is involved and consequently how to prevent it;
- Obtaining the oxidative impurities that are formed under accelerated and long-term storage conditions as information that can be used to develop appropriate stability-indicative chromatographic methods [29];
- Controlling genotoxic impurities;
- A better, more detailed understanding of a drug.

### 5.1. Autoxidation

**Figure 12:**
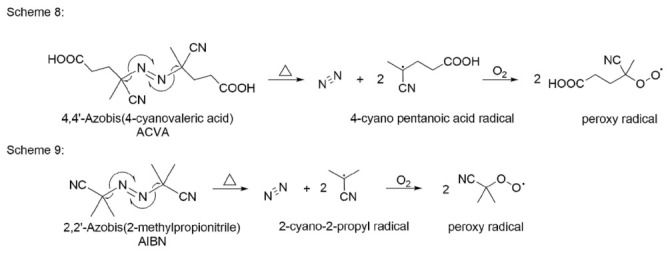
4,4′-Azobis(4-cyanovaleric acid) and 2,2′-azobis(2-methylpropionitrile) mechanism of decomposition and reactions to peroxy radicals.

As described in the autoxidation section, peroxy radicals (ROO˙) are the driving force behind these reactions. Azo compounds are, therefore, the best choice here, because peroxy radicals are formed during thermal decomposition in solution. The reaction in Figure 12 shows the formation of peroxy radicals from 4,4′-azobis(4-cyanovaleric acid) and 2,2′-azobis(2-methylpropionitrile). In practice, the use of both of these azo compounds depends on the solutions that are used, whereby 4,4′-azobis(4-cyanovaleric acid) is more water soluble than 2,2′-azobis(2-methylpropionitrile) [101,102].

Upon heating these azo compounds, nitrogen is released and carbon-centered radicals are formed. These unstable radicals are rapidly oxygenated in an oxygen-saturated solution. The final product is a peroxy radical, which serves as a radical initiator in the oxidation of a drug [29].

The molar ratio of the azo compounds to the drug is usually around 10:1. The possibility of disproportionation of peroxy radicals should also be emphasized at this point, whereby alkoxy radicals can be formed, which are less selective than peroxy radicals. This results in poorer selectivity, which can lead to misinterpretation of data. One of the possible solutions for the disproportionation problem is the use of at least 10% methanol in acetonitrile–water solvents, as this suppresses the activity of the alkoxy radicals that are formed [32].

### 5.2. Nucleophilic/Electrophilic (Peroxide Mediated)

These peroxide-mediated reactions can be confirmed using a solution of hydrogen peroxide. The critical experimental parameters in this case are temperature, solvent composition, and hydrogen peroxide concentration [103,104]. Even at room temperature, the reaction with hydrogen peroxide is rapid, so there is no need to raise the temperature, although the possibility for the formation of hydroxyl radicals increases at higher temperatures. For the solvent mixture, it is important to add cosolvents to water, such as methanol or acetonitrile, as both of these quench the hydroxyl radicals. Another consideration is the pH of the solution, as the reaction between amine and hydrogen peroxide is slowed greatly if nitrogen is protonated. Therefore, it is necessary to control the pH for compounds with amine groups [105].

### 5.3. Transition Metal Ions

Transition metal complexes can catalyze oxidation in a variety of ways. Heterolytic reactions with metal complexes can be divided into three groups:

- Reactions with hydroperoxides;
- Activation of molecular oxygen;
- Direct reaction of metal complexes with the substrate.

In the study of single electron transfer to dioxygen, the primary interest is the last of these mechanisms. Transition metal complexes of iron (III) and copper (II) are usually used in degradation studies as their redox potentials are not excessively positive, on the basis that the electron transfer mechanism generally depends on the ionization potential of the substrate [106].

Iron (III) and copper (II) are not usually involved in mechanism 2, as long as no source of reducing equivalents is present and mechanism 1 is by-passed by the use of a peroxide-free solvent. With this in mind, the oxidation mechanism during metal ion testing can generally be interpreted as an electron transfer mechanism [107,108].

## 6. New Stressors for Accelerated Oxidation Studies

Oxidation in multicomponent systems can be complex due to various factors, such as the microenvironment, oxygen diffusion, crystal habits, plasticization by excipients, salt formation, and others. Furthermore, all oxidative susceptibility tests that are described above are performed in solution. The impurities that are formed in solutions might not always be the same as in the final products, which, in the case of the majority of pharmaceutical products, is a solid [86,109,110].

Compounds that are susceptible to oxidation undergo various tests during the development of their formulation. One of these is the spiking of impurities into drug–excipient mixtures [91], which greatly simplifies the microenvironment compared to real formulations. In the final formulations, spiking is more difficult to perform, as it should be performed most often in the granulation process so that the impurity is evenly distributed throughout the formulation.

One of the most recently described oxidative stressors for forced degradation in solids is the PVP–hydrogen peroxide (PVP–H_2_O_2_) complex. PVP is one of the most commonly used tablet binders, so Modhave et al. formed a complex with H_2_O_2_ for solid-state oxidation testing [111]. There are also other polymers that are suitable for forming complexes, such as cross-linked PVP, soluplus, and others.

Modhave et al. prepared and characterized the PVP–H_2_O_2_ complex. For the preparation, 12 mg PVP powder was added to 18 mL precooled (in an ice bath) 30% (*w*/*w*) H_2_O_2_ solution in a glass beaker and the solution was stirred for one hour. The resultant mixture was left at 25 °C for 15 h. Then, the sample was dried in a desiccator (under a vacuum) at 40 °C for 35 days. The solid powder that was obtained after the drying was crushed using a mortar and pestle with liquid nitrogen. The solid PVP–H_2_O_2_ powder that was obtained was stored at 2 °C to 8 °C. Vortioxetine hydrobromide and PVP–H_2_O_2_ were then compressed into compacts and exposed to stress conditions (40 °C; 75% relative humidity). Over time, both the color and shape of the compacts changed. After 10 days under accelerated conditions, the oxidative impurity content was analyzed. The amount of degradation product that was formed was approximately twice as high (6%) under the open conditions compared to closed conditions. This can be explained by the direct exposure to moisture absorption by the PVP–H_2_O_2_ complex and the release of free H_2_O_2_. It should be noted here that most of the impurity was formed within the first day and was then constant until the end of the experiment. This suggests that the stationary state of the solid-state reaction kinetics was reached within one day. A 1:1 ratio (drug to PVP–H_2_O_2_) was used to make the compacts, however, a ratio in favor of the excipient would be more realistic. With the high content of PVP–H_2_O_2_, they achieved the maximum formation of the degradation impurity [111]. It would be interesting to prepare mixtures of different ratios of the complex and PVP and to study how the content of PVP–H_2_O_2_ influences the extent of drug degradation. Additionally, it would be prudent to determine the amount of peroxides in the mixture prior to the preparation of the compacts with the drug.

Another interesting approach for peroxide-mediated oxidation stress testing that was described recently was with urea–hydrogen peroxide. This is a complex that undergoes solid-state decomposition at elevated temperatures (30 °C and higher) and releases hydrogen peroxide vapor. This study was carried out in two open vials that contained the urea–hydrogen peroxide and the tested pharmaceutical solid. [112].

Compared to the PVP–H_2_O_2_ complex, hydrogen peroxide from the urea complex reacts only with the exposed surface of the drug. In contrast, PVP–H_2_O_2_ is more homogeneously mixed with the drug. Moreover, better contact between the hydrogen peroxide and the drug was achieved. The PVP–H_2_O_2_ complex appears to be more suitable for use in stress testing. PVP–H_2_O_2_ is not ideal though, because it is very susceptible to moisture which changes the shape and color of the compacts. It would certainly be interesting to compare both stressors on the same drug with an in-depth comparison of the pros and cons of each. Nevertheless, the use of these new stressor methodologies provides useful information regarding the oxidative susceptibility and potential degradation products in the solid state, and this information can be obtained in a short period of time. The successful implementation of such screening methods is expected to greatly facilitate both analytical method development and formulation design.

## 7. Conclusions

Oxidation is the second most common degradation reaction in pharmaceutical products after hydrolysis. Therefore, it is important that oxidation reactions are well understood at each step of the development of a new product. Forced degradation studies provide the first information about the stability and possible degradation pathways of a drug. At this point, it is important to determine the structure of the degradation products, which is nowadays mostly done by liquid chromatography–high resolution mass spectrometry. The structures of degradation products enlighten the underlying oxidation mechanisms. However, certain degradation products that are detected under forced degradation might not be formed in the drug formulation. Therefore, the aim and focus of these studies is to obtain as similar degradation products as possible to those that are encountered under nonaccelerated storage conditions. The knowledge that is obtained on the mechanisms helps in the identification of critical parameters that need to be controlled to prevent the occurrence of these reactions in the final product. If a particular compound turns out to be susceptible to oxidation during forced degradation tests, then oxidation, in particular, can be focused upon more intensely with the use of additional approaches to study solid oxidation, which can then provide better insight into the actual oxidative susceptibility. Compounds that are susceptible to oxidation can react with impurities in their excipients, especially peroxides. However, if there are no good alternatives to excipients with peroxide impurities, their impact can be reduced by the addition of appropriate antioxidants. However, such decisions should be based on a knowledge of the degradation pathways that can be attained through rigorous degradation studies.
